# Fat mass affects nutritional status of ICU COVID-19 patients

**DOI:** 10.1186/s12967-020-02464-z

**Published:** 2020-08-03

**Authors:** Antonino De Lorenzo, Maria Grazia Tarsitano, Carmela Falcone, Laura Di Renzo, Lorenzo Romano, Sebastiano Macheda, Anna Ferrarelli, Demetrio Labate, Marco Tescione, Federico Bilotta, Paola Gualtieri

**Affiliations:** 1 Section of Clinical Nutrition and Nutrigenomics, Department of Biomedicine and Prevention, University of Rome Tor Vergata, 00133 Rome, Italy; 2 Department of Experimental Medicine, University of Rome Sapienza, 00161 Rome, Italy; 3 Unit of Radiology, Grande Ospedale Metropolitano Bianchi Melacrino Morelli, 89124 Reggio Calabria, Italy; 4 School of Specialization in Food Science, University of Rome Tor Vergata, 00133 Rome, Italy; 5 Unit of Intensive Care Medicine and Anesthesia, Grande Ospedale Metropolitano Bianchi Melacrino Morelli, 89124 Reggio Calabria, Italy; 6 Department of Anesthesiology and Critical Care, University of Rome Sapienza, 00161 Rome, Italy

**Keywords:** Fat mass, Covid-19, BMI, Obesity, ICU, PNI, Steatosis

## Abstract

**Background:**

Obesity and steatosis are associated with COVID-19 severe pneumonia. Elevated levels of pro-inflammatory cytokines and reduced immune response are typical of these patients. In particular, adipose tissue is the organ playing the crucial role. So, it is necessary to evaluate fat mass and not simpler body mass index (BMI), because BMI leaves a portion of the obese population unrecognized. The aim is to evaluate the relationship between Percentage of Fat Mass (FM%) and immune-inflammatory response, after 10 days in Intensive Care Unit (ICU).

**Methods:**

Prospective observational study of 22 adult patients, affected by COVID-19 pneumonia and admitted to the ICU and classified in two sets: (10) lean and (12) obese, according to FM% and age (De Lorenzo classification). Patients were analyzed at admission in ICU and at 10th day.

**Results:**

Obese have steatosis, impaired hepatic function, compromise immune response and higher inflammation. In addition, they have a reduced prognostic nutritional index (PNI), nutritional survival index for ICU patients.

**Conclusion:**

This is the first study evaluating FM% in COVID-19 patient. We underlined obese characteristic with likely poorly prognosis and an important misclassification of obesity. A not negligible number of patients with normal BMI could actually have an excess of adipose tissue and therefore have an unfavorable outcome such as an obese. Is fundamental personalized patients nutrition basing on disease phases.

## Background

Obesity, measured as body mass index (BMI), is reported to associate with increase the risk of developing severe pneumonia in COVID-19 [1]. Indeed, the risk correlated to obesity with COVID-19 severity is greater in metabolic associated fatty liver patients [2]. At the basis, we found altered mechanism of inflammation and immune response typic of obesity and correlated with alteration in the levels of circulating cytokines [3]. In particular, obese patients have higher concentrations of TNF-alpha, MCP-1 and IL-6 which are produced by visceral and subcutaneous adipose tissue and implicated in innate immunity [4, 5].

Furthermore, adipose tissue releases high levels of leptin, which creates an unfavorable inflammatory milieu that leads to dysregulation of the immune response [6].

In 2009, during H1N1 pandemia, in obese patients it was characterized changing of differentiation of B cells [7, 8], predisposing to a greater risk of contracting influence, but also of being more contagious towards other people [9]. However, they have impaired memory T cell response and vaccination efficacy [10]. Specifically, reduced response of virus-specific CD8 + lymphocytes and suboptimal macrophage functionality have been demonstrated, which could explain the low response to the vaccine stimulus [11].

For COVID-19, in Intensive Care Unit (ICU), it was observed that the highest percentage are patients affects by severe obesity, with BMI > 35 kg/m^2^ [12].

The BMI does not reflect necessarily the fat mass (FM). There are evidence that suggest how FM% rather than BMI, predicts inflammatory (TNF-alpha, MCP-1 and IL-6) and immune (leptin) response. These ultimate variables that relates with immune and inflammatory response in FM% [13].

Despite the relationship between FM% and severity of progression inflammatory response in patients admitted to ICU for underlying infective disease, there are no available data on the relevance of FM% in COVID-19 patients treated in ICU.

Moreover, prognostic nutritional index (PNI) can be used to evaluate the nutritional status and survival for ICU patients [14].

Aim of this prospective observational study, in patients admitted to ICU for COVID-19, is to evaluate the relationship between FM% and immune-inflammatory response, after 10 days in ICU.

Also, we want to investigate the metabolic associated fatty liver and PNI and the comparison between FM% and BMI.

## Subjects and methods

After IRB approval (Regional Ethic Committee, Section “Area Sud”, 20th April, 2020) and having obtained the signed informed consent by the next of kin, clinical and anthropometric data of patients with COVID-19 pneumonia admitted to the ICU of the Hospital "Bianchi Melacrino Morelli" Reggio Calabria, Italy between 13 March and 6 April 2020, were recorded and analyzed. Standard therapeutic protocol included, for all patients: low molecular weight heparin (LMWH), azithromycin, hydroxychloroquine, lopinavir/ritonavir.

Prospective observational study of 22 adult patients affected by COVID-19 pneumonia and admitted to the ICU. Patients with a history of neutropenia, acquired immunodeficiency, who underwent transplants or who received previous immunosuppressive therapies were excluded.

Rather than considering only the BMI, patients recruited in this study were categorized in two sets: “lean” or “obese” according to FM% and age, based on criteria presented by De Lorenzo [15].

Computed tomography (CT) (GE Medical SYSTEMS, Gamma Optima, USA) without intravenous contrast was performed within 24 h of admission to the emergency department.

Hepatic steatosis was evaluated on CT images in 4 liver segments, independently, by 2 qualified operators (LR and CF) [16]. Liver Spleen Ration (LRS) was calculated If reported difference between the measures were > 5%, a third operator was asked to repeat the evaluation of CT. From CT chest image, waist circumference was measured at the last rib with distance measurement tools. Where part of the abdomen was outside the field of the image, waist circumference was estimated with a continuous arc [16]. To estimate the FM% we used the Siri Eq. [17]. The subcutaneous fat thickness was measured at CT, given the agreement between the CT and plicometry method [18]. Body density was obtained by the equation of Durnin, using two subcutaneous fat thicknesses of the chest, suprascapular and suprailiac and the correction factors according to age, sex and folds used [19]. All CTs were performed with patients in the supine position with arms folded and hands positioned under neck. The subscapular fat thickness was measured in cross section starting from the origin of the scapular spine on the posterior medial edge up to the skin. The suprailiac fat thickness was measured in cross Sect. 2 cm from the last rib on the middle axillary line up to the skin. The subcutaneous fat thickness parameters were measured two times on CT. It was used the mean value for equation of Durnin [18]. Only at baseline, CT was used to estimated FM% to stratificate the sample. The BMI was calculated as weight (kg) divided by height (m) squared and the patients were classified as follows: obese (OB) for BMI > 29.99 kg/m^2^, pre obese (PO) for BMI between 25.00 kg/m^2^ and 29.99 kg/m^2^, normal weight (NW) for BMI between 18.50 and 24.99 kg/m^2^.

Prognostic nutritional index (PNI) was calculated for each patient as a serum albumin (g/dL) × 10 + total lymphocyte count (mm − 3) × 0.005 [20].

The following blood analysis were performed: C-reactive protein (mg/L), glycemia (mg/dL), creatinine (mg/dL), albumin (g/dL), AST (U/L), ALT (U/L), indirect bilirubin (mg/dl), total bilirubin (mg/dl), direct bilirubin (mg/dl), Platelets (10^3^/μL), white blood cells (WBC) (10^3^/μL), neutrophils (10^3^/μL), Lymphocytes (10^3^/μL), PNI, fibrinogen (mg/dL), D-dimer (ng/mL).

### Statistics

Calculation of the sample size was based on a comparison between matched pairs, a power of 80%, a significance level of 5% (two-tails) and the detection of an effect size of 0.6 between the pairs. According to the study setting, necessary calculated sample size is 20 patients and the G*Power software (Version 3.1.9.6, Germany) was used. [21] Since 20% of the sample may not have all the expected parameters, 24 subjects were enrolled.

All statistical analyzes were conducted with SPSS 23 software (version 23.0, IBM, Armonk, NY, USA). The data collected before statistical evaluations were analyzed for the presence of outliners and for non-normally distribution with the Kolmogorov–Smirnov Test. The categorical variables have been reported in percentage, while the continuous ones as median and interquartile range. Before, the differences between lean and obese subjects were assessed at admission with the Mann Whitney test for independent samples. Subsequently, the differences in lean and obese subjects were assessed between admission and 10th day with the Wilcox test for matched pairs. Cohen’s Kappa was used with binary data to measure the agreement between adiposity classification according to the FM% criterion and the BMI. According to Landis and Koch [22], Cohen’s Kappa (κ) values could indicate an agreement: poor (κ < 0.00), light (0.00 ≤ κ ≤ 0.20), discrete (0.21 ≤ κ ≤ 0.40), moderate (0.41 ≤ κ ≤ 0.60), substantial (0.61 ≤ κ ≤ 0.80) or near-perfect (κ > 0.80). Furthermore, the false-positive rate and false-negative rate were calculated for the different classification methods.

Correlation analysis was conducted with Spearman's rho test. Statistical significance was set to a value of *p* < 0.05. All *p* values shown are two-tailed.

## Results

**Fig. 1:**
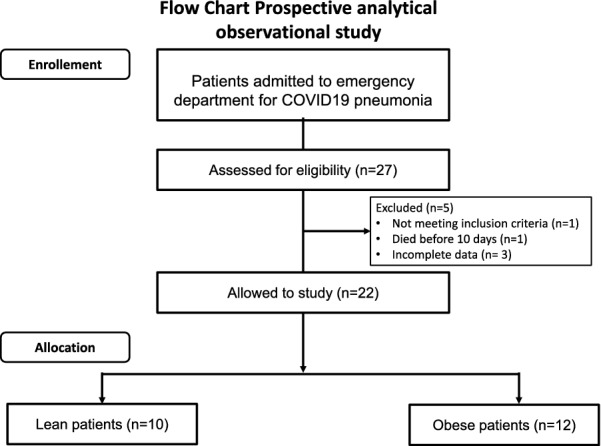
Flow chart prospective analytical observational study

A total of 27 patients were evaluated for this prospective analytical observational study, 5 subjects were excluded from the study because the following reasons: 1 because COVID-19 negative, 1 died before 10th day; 3 had incomplete data. Finally, 22 patients were included in the study (Fig. 1). Mean age of enrolled patients was 58 years (range 49–67), 45% were females and 55% males. Patients were divided into two groups according to FM% and age: 12 patients were “obese” and 10 were “lean” (Table 1).

**Table 1:** Descriptive and compared between group at baseline

	Overall (n 22)	Lean (n 10)	Obese (n 12)	*p*
	Median (IQR)	Median (IQR)	Median (IQR)	
Anthropometrics and body parameters				
Age (years)	58.50 (49.00; 67.75)	57.00 (47.00; 68.00)	65.00 (52.50; 67.50)	0.49
Subscapular Thickness (mm)	17.51 (11.83; 21.39)	11.30 (9.74; 16.30)	21.34 (17.94; 26.64)	0.001
Suprailiac Thickness (mm)	21.11 (12.00; 25.51)	11.60 (6.70; 16.30)	25.00 (21.23; 28.05)	0.004
Sum Thickness (mm)	38.96 (23.91; 46.32)	23.10 (18.00; 32.80)	46.03 (41.21; 49.21)	0.001
Fat Mass (%)	37.81 (27.71; 46.52)	27.57 (22.49; 33.18)	45.68 (40.26; 47.59)	0.001
Waist Circumference (cm)	99.49 (96.23; 113.34)	97.11 (94.22; 99.50)	101.25 (99.10; 116.90)	0.048
Liver Attenuation (HU)	47.94 (43.04; 51.34)	51.46 (49.94; 54.14)	44.37 (37.13; 47.94)	0.005
Spleen Attenuation (HU)	51.23 (44.50; 54.14)	54.30 (49.45; 57.26)	51.70 (48.10; 55.89)	0.791
LSR Attenuation	0.92 (0.82; 1.00)	0.96 (0.82; 1.06)	0.82 (0.75; 0.88)	0.017
Blood chemistry parameters				
C-reactive Protein (mg/L)	74.30 (55.20; 113.00)	88.60 (45.18; 112.00)	89.20 (60.5; 107.00)	0.728
Glycemia (mg/dL)	111.00 (97.00; 138.00)	111.00 (96.00; 134.00)	113.00 (96.93; 146.25)	0.855
Creatinine (mg/dL)	0.80 (0.60; 1.09)	0.94 (0.74; 1.33)	0.70 (0.60; 0.90)	0.173
Albumin (g/dL)	3.30 (3.00; 4.05)	3.50 (2.78; 4.03)	3.30 (3.10; 4.30)	0.830
AST (U/L)	39.00 (31.00; 56.50)	33.00 (28.00; 39.25)	55.00 (36.00; 81.00)	0.027
ALT (U/L)	29.00 (16.50; 60.50)	18.00 (12.75; 36.00)	41.00 (21.00; 96.00)	0.034
Indirect bilirubin (mg/dl)	0.71 (0.44; 1.15)	0.80 (0.50; 1.08)	0.58 (0.26; 1.61)	0.431
Total bilirubin (mg/dl)	1.00 (0.77; 1.40)	1.00 (0.90; 1.40)	0.92 (0.52; 2.40)	0.431
Direct bilirubin (mg/dl)	0.36 (0.26; 0.41)	0.40 (0.32; 0.41)	0.31 (0.22; 0.94)	0.352
Platelets (10^3^/μL)	187.00 (132.75; 301.25)	202.00 (141.00; 363.00)	172.00 (120.00; 285.00)	0.634
WBC (10^3^/μL)	6.65 (4.67; 11.33)	5.17 (3.79; 8.73)	6.99 (4.83; 13.07)	0.223
Neutrophils (10^3^/μL)	4.04 (3.16; 7.08)	3.78 (3.04; 7.24)	4.33 (3.47; 8.61)	0.491
Lymphocytes (10^3^/μL)	0.85 (0.55; 1.21)	0.89 (0.39; 1.53)	0.81 (0.65; 1.00)	0.711
PNI	36.01 (30.00; 44.51)	35.00 (27.75; 40.26)	36.00 (31.00; 41.0)	0.475
Fibrinogen (mg/dL)	527.00 (414.00; 680.01)	592.00 (390.50; 677.50)	521.00 (390.00; 738.50)	0.953
D-Dimer (ng/mL)	387.00 (224.00; 852.50)	336.00 (164.50; 1974.00)	438.00 (255.00; 869.00)	0.549

Differences among groups at baseline. All parameters are presented as median (interquartile range) and were compared by Mann Whitney test Statistical significance was attributed as *p* < 0.05

*IQR* Interquartile Range, *LSR* Liver Spleen Ratio *AST* Aspartate Aminotransferase, *ALT* Alanine Aminotransferase, *WBC* White Blood Cell, *PNI* Prognostic Nutritional Index

**Fig. 2:**
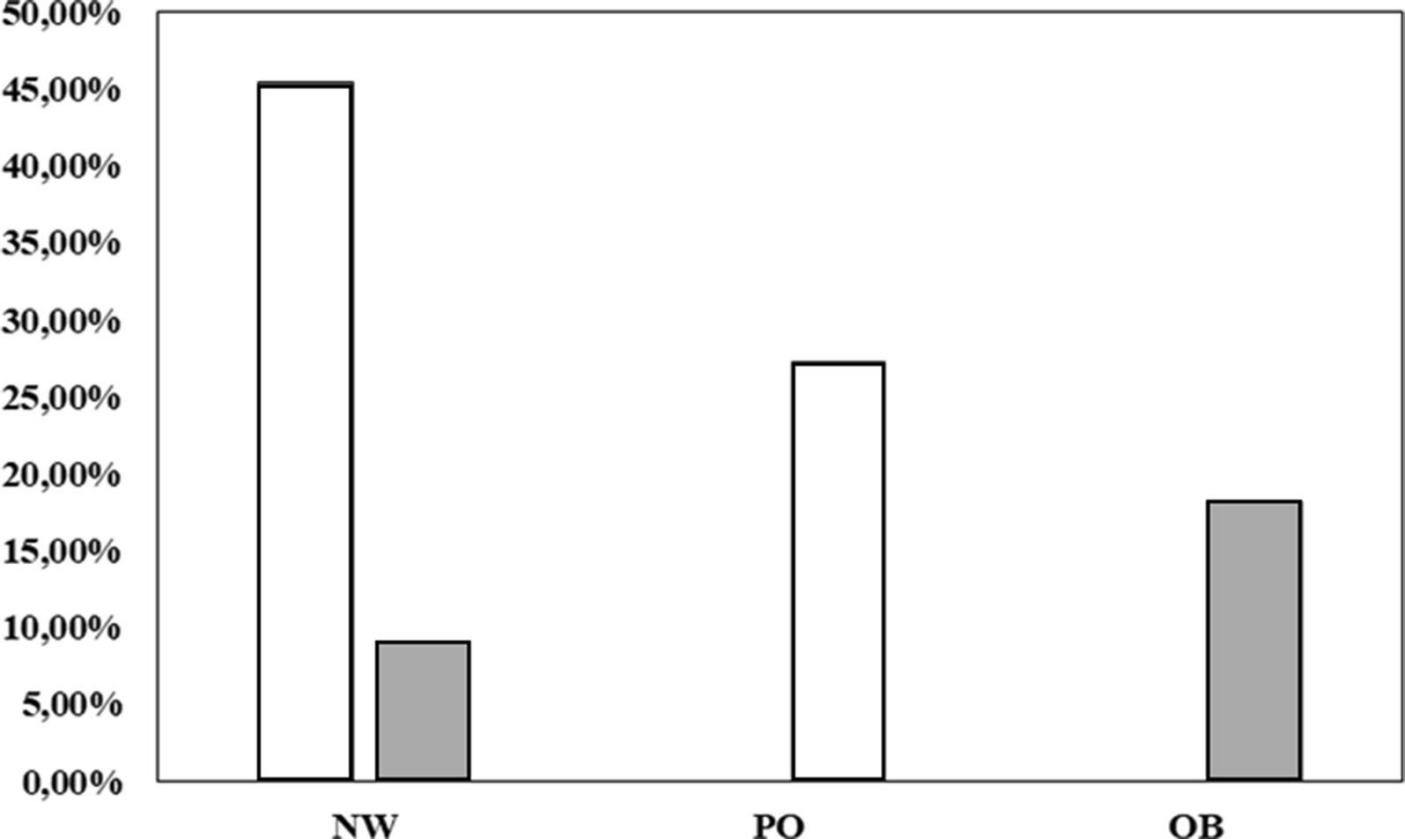
Baseline characteristics of “lean” vs. “obese” patients

The BMI-based classification, as compared to FM%, presents a discrete and significant Cohen κ-value (κ = 0.405 *p* < 0.000). According to the BMI, 36% of the patients were categorized differently, in detail, 9% of the patients that presented a BMI within normal values were detected to have a FM% criteria for being considered “obese” according to FM% and age based on criteria presented by De Lorenzo [15] and 27% of the patients classified PO were “obese”. (Table 2) (Fig. 2). The FM%-based categorization coincided to BMI-based categorization for the 10 patients considered “lean” according the FM% and for 4 patients considered “obese”. Lastly, for this sample the frequency of false negatives was 36%, while there were no false positives.

**Table 2:** Contingency table, κ coefficient of BMI for indicating the misclassification of obesity in population sample

		FM% classification		
		Lean	Obese	Total
BMI Classification	NW (18.50–24.99)	45.40% (10)	9.10% (2)	54.50% (12)
	PO (25.00–29.99)	0.00% (0)	27.30% (6)	27.30% (6)
	OB (≥ 30.00)	0.00% (0)	18.20% (4)	18.20% (4)
	Total	45.40% (10)	54.60% (12)	100.00% (22)
	κ^*^ = 0.405			

All values are presented as percentage (numbers) and κ have *p values were < 0.001

*FM* Fat Mass, *BMI* Body Mass Index, *NW* Normal Weight, *PO* Pre-Obese, *OB* Obese

Baseline characteristics of “lean” vs. “obese” patients. No statistical differences were present for age and spleen attenuation between groups. Liver and LSR attenuation were lower in “obese” than in “lean” (Table 1). The ALT and AST were significantly more elevated in “obese” than in “lean”. No other statistical difference was found in blood chemistry parameters between the groups. At day 10th, the C-reactive protein, direct bilirubin, fibrinogen concentrations were lower in were lower than in “lean” (respectively *p* = 0.005; *p* = 0.033; *p* = 0.028), the lymphocytes concentration was increased in “lean” (*p* = 0.038) and not changed was observed in “obese”. Comparison of baseline to day 10th, concentrations of albumin and PNI decreased in “obese” while remained unchanged in “lean” patients. No statistical difference was present in “lean” and “obese” groups between baseline and 10 days (Table 3).

**Table 3:** Difference between baseline and 10th day for each group

Blood chemistry parameters	Lean (n 10)			Obese (n 12)		
	Baseline	10th day		Baseline	10th day	
	Median (IQR)	Median (IQR)	*p*	Median (IQR)	Median (IQR)	*P*
C-reactive Protein (mg/dL)	88.60 (45.18; 112.00)	17.65 (8.29; 30.20)	0.005	89.20 (60.5; 107.00)	54.95 (27.73; 103.35)	0.465
Glycemia (mg/dL)	111.00 (96.00; 134.00)	103.50 (92.50; 127.25)	0.109	113.00 (96.93; 146.25)	108.00 (87.00; 133.00)	0.345
Creatinine (mg/dL)	0.94 (0.74; 1.33)	1.00 (0.80; 1.89)	0.180	0.70 (0.60; 0.90)	0.71 (0.60; 0.96)	0.206
Albumin (g/dL)	3.50 (2.78; 4.03)	3.15 (2.53; 3.48)	0.109	3.30 (3.10; 4.30)	3.00 (2.80; 3.20)	0.018
AST (U/L)	33.00 (28.00; 39.25)	26.00 (18.50; 34.25)	0.109	55.00 (36.00; 81.00)	41.00 (21.00; 78.00)	0.176
ALT (U/L)	18.00 (12.75; 36.00)	37.00 (15.75; 57.50)	0.285	41.00 (21.00; 96.00)	51.00 (29.00; 93.01)	0.866
Indirect bilirubin (mg/dl)	0.80 (0.50; 1.08)	0.46 (0.31; 0.60)	0.176	0.58 (0.26; 1.61)	0.63 (0.36; 1.87)	0.285
Total bilirubin (mg/dl)	1.00 (0.90; 1.40)	0.57 (0.50; 0.80)	0.091	0.92 (0.52; 2.40)	1.15 (0.70; 2.61)	0.285
Direct bilirubin (mg/dl)	0.40 (0.32; 0.41)	0.18 (0.11; 0.23)	0.033	0.31 (0.22; 0.94)	0.55 (0.25; 0.82)	1.000
Platelets (10^3^/μL)	202.00 (141.00; 363.00)	216.00 (185.00; 378.00)	0.612	172.00 (120.00; 285.00)	263.00 (180.00; 333.50)	0.139
WBC (10^3^/μL)	5.17 (3.79; 8.73)	6.22 (3.49; 8.89)	0.866	6.99 (4.83; 13.07)	4.55 (3.62; 10.05)	0.173
Neutrophils (10^3^/μL)	3.78 (3.04; 7.24)	4.24 (2.70; 6.72)	0.866	4.33 (3.47; 8.61)	2.67 (1.72; 5.62)	0.214
Lymphocytes (10^3^/μL)	0.89 (0.39; 1.53)	1.41 (0.40; 2.20)	0.038	0.81 (0.65; 1.00)	1.01 (0.50; 1.15)	0.176
PNI	35.00 (27.75; 40.26)	32.51 (25.25; 34.76)	0.109	36.00 (31.00; 41.0)	30.01 (28.00; 32.01)	0.022
Fibrinogen (mg/dL)	592.00 (390.50; 677.50)	265.00 (210.00; 358.00)	0.028	521.00 (390.00; 738.50)	499.00 (208.00; 634.00)	0.593
D-Dimer (ng/mL)	336.00 (164.50; 1974.00)	367.01 (250.00; 4380.50)	0.144	438.00 (255.00; 869.00)	435.00 (304.75; 521.50)	0.161

Differences between baseline and 10th day for each group. All parameters are presented as median (interquartile range) and were compared by Wilcoxon test. Statistical significance was attributed as *p* < 0.05

*IQR* Interquartile Range, *AST* Aspartate Aminotransferase, *ALT* Alanine Aminotransferase, *WBC* White Blood Cell, *PNI* Prognostic Nutritional Index

Finally, an inversely proportional correlation was observed between FM% and liver attenuation (correlation coefficient = − 0.702 and *p* = 0.002).

## Discussion

The distinctive feature of this prospective observational study, in patients admitted to the ICU for COVID-19, is a persistent lymphocyte reaction at 10th day in FM%-based “obese” patients that suggest a protract inflammatory reaction. In these patients, at the beginning of ICU treatment, the metabolic associated fatty liver, PNI and the immune-inflammatory response are severely compromised. Furthermore, at baseline more patients evaluated according FM% result to be “obese” than using the BMI criteria.

In COVID-19 patients it is crucial to find risk factors associated with worse clinical course to allocate appropriate resources. However, population characteristics are fundamental for prognosis. In Italy, COVID-19 mortality is strongly influenced by different comorbidities [23] and 52% of deaths are above 80 years of age, unlike China, for which only 20% are above the same age threshold [23].

In particular, pre-existing pathologies including obesity, cardiovascular co-morbidity, arterial hypertension and type 2 diabetes mellitus are established risk factors [24–26]. Obesity also can be associated to insulin-resistance, that alters immune response [27]. Obese patients have greater infectivity correlated with exhalation, since they have higher ventilator volumes, due to a lower expansion capacity of the thoracic cavity, which consequently limits the lung expansion [28]. This also results in increased aerosol production [28]. In particularly, Maier et al. [29] showed that obese patients have a longer viral interaction.

In the case of COVID-19, it has been observed that the infectious charge has an average duration of 20 days, but it can last up to 37 days after the infection [30]. It remains to be shown the pathophysiological characteristics of patients who have a contagious duration of up to 37 days.

Hence, the importance of assessing the inflammatory state, through circulating cytokines, has already been highlighted in patients suffering from acute respiratory distress syndrome. Indeed, it was possible to identify two distinct phenotypes, with two different mortality risks. This is fundamental for indicating the patient future prognosis [31]. In obese, lipid metabolism is already altered [13, 32] and a COVID-19 infection leads to an overexpression of the genes involved with a further increase in the production of pro-inflammatory cytokines and a reduced capability responding to infection.

In our study, “lean” patients-according FM%, showed a significative reduction in C-protein reactive, direct bilirubin and fibrinogen and an important increase of lymphocytes at day 10th of ICU. Increased FM% is associated with a reduced ICU treatment response. Actually, our data showed that “obese” do not show the same improvement, based on biochemical-clinical parameters, respect to “lean” after the first 10th ICU days.

According to presented data, adipose tissue quantity acts on therapeutic goal achievement. Increased adipose tissue leads to a lipid metabolism modification with increased storage of fat in liver and onset of steatosis in “obese”. Consequently, these patients suffer for high production of pro-inflammatory cytokines [33, 34] and conduct to unfavorable condition, requiring defined protocols to counter malnutrition resulting [35].

Additionally, these patients are at higher risk for infection also because of COVID-19 use angiotensin converting enzyme 2 (ACE2) receptors to enter the host cell [36]. The ACE2 is expressed in different tissues: kidney, lung, heart and adipose tissue [36]. COVID-19 infection leads to an upregulation of the genes associated with lipid metabolism, involved in the regulation of inflammation [37].

Thus, obese have a higher expression of ACE2 and are therefore more susceptible to this infection [38]. The complex picture is characterized by increased predisposition to infection and reduced ability to respond to it. In addition, these patients already present organ damage that induces worse response to treatments.

According to the results, the main aim is a proper nutritional medical therapy, which takes into account the amount of fat, as a risk factor for complications in COVID-19. Therefore, the therapeutic approach must be customized on the body composition. In addition, the loss of body protein content is a negative prognosis factor and it has been a constant observation in ICU.

A further aim, in the not-affected, affected and discharged COVID-19 patients, is the saving and recovery of lean body mass, following an appropriate protein prescription.

In pre-COVID-19 patients, a personalized and balanced Italian Mediterranean Reference Diet characterized by anti-inflammatory and antioxidant properties [39], should be adopted as obesity preventive and therapeutic tool. The protein intake required is based on lean mass content (2 g/kg of lean mass/day), a parameter that can be directly measured or calculated with prediction equations, accessible to all users [15].

In COVID-19 patients, a macronutrient balance calculated according to the clinical condition, a correct calorie intake based on the metabolic condition and all micronutrients must be guaranteed. In detail, respiratory failure requires hyperlipidic nutritional medical therapy, to counter hypercapnia and promoting metabolic flexibility [35, 40]. The calorie prescription must be adjusted daily, following the catabolic and anabolic phases of hospitalization. Similarly, the protein prescription must be modulated according to the metabolic phase. In the anabolic phase, the protein administrated should not be counted in the daily energy expenditure and the protein intake must be 1.3 g/kg of body weight/day [40].

In post-COVID-19 patients, keeping in mind that the fragility deriving from bedrest and inadequate nutrition, due to the ventilatory support, a specific nutritional and motor rehabilitation must be provided [40]. For patients with comorbidity, nutrition support to anabolic and recovery stress represent a complex passage. Diet therapy, personalized based on the body composition [35], must be hyperproteic, 2–2.5 g/kg of lean mass/day, complete with all amino acids and enriched with branched amino acids, to promote anabolism. The meals consistency must be progressively personalized according to the subject ability to feed.

Our data, even if the sample size required by statistical tests is respected, nevertheless presents a limited number of patients. Another limit was FM% estimate. At the same time, it is a strength, which has allowed an estimate of body composition, since other methods such as bioimpedance and anthroplicometry are difficult to apply in ICU.

It is hoped that from the COVID-19 lesson, the Public Institutions will promote the prevention and treatment of obesity and sarcopenia, through healthy nutrition and a correct lifestyle.

The comorbidities costs and the obstacle in the clinical treatment of an obese patient, in addition to the known health-care costs [41], has been paid with human lives.

## Conclusions

Our data originally demonstrate that FM% and not only BMI correlates with the course of COVID-19 patients admitted to ICU. Of note a not negligible number of patients with normal BMI could actually have an excess of adipose tissue and therefore have an unfavorable outcome such as an obese [42]. Since it is the actual representation of adipose tissue that is the driver of pro-inflammatory modulation the FM% might represent a better assessment tool than BMI.

Future studies are required to determine the relevance of FM% in predicting the clinical outcomes of COVID-19 patients and might be a useful approach also for out of hospital surveillance.

## Data Availability

The datasets used and/or analyzed during the current study are available from the corresponding author on reasonable request.

## Abbreviations

ACE2: Angiotensin converting enzyme 2
BMI: Body mass index
CT: Computed tomography
FM: Fat mass
ICU: Intensive Care Unit
LSR: Liver spleen ratio
NW: Normal weight
PNI: Prognostic nutritional index
PO: Pre obese
OB: Obese
